# The rapid replacement of the Delta variant by Omicron (B.1.1.529) in England

**DOI:** 10.1126/scitranslmed.abo5395

**Published:** 2022-05-03

**Authors:** Robert S. Paton, Christopher E. Overton, Thomas Ward

**Affiliations:** Data Science and Analytics, UK Health Security Agency, Nobel House, London, UK, SW1P 3JR

## Abstract

The emergence of the B.1.1.529 (Omicron) variant caused international concern due to its rapid spread in Southern Africa. It was unknown whether this variant would replace or co-exist with (either transiently or long-term) the then-dominant Delta variant on its introduction to England. We developed a set of hierarchical logistic growth models to describe changes in the frequency of *S* gene target failure (SGTF) PCR tests, which was a proxy for Omicron. The doubling time of SGTF cases peaked at 1.56 days (95% CI: 1.49, 1.63) on the 5^th^ of December, while triple positive cases were halving every 5.82 days (95% CI: 5.11, 6.67) going into Christmas 2021. We were unable to characterize the replacement of Delta by Omicron with a single rate. The replacement rate decreased by 53.56% (95% CrI: 45.38, 61.01) between the 14^th^ and 15^th^ of December, meaning the competitive advantage of Omicron approximately halved. Preceding the changepoint, Omicron was replacing Delta 16.24% (95% CrI: 9.72, 23.41) faster in those with two or more vaccine doses, indicative of vaccine escape being a substantial component of the competitive advantage. Despite the slowdown, Delta had almost entirely been replaced in England within a month of the first sequenced domestic case. The synchrony of changepoints across regions at various stages of Omicron epidemics suggests that the growth rate advantage was not attenuated due to biological mechanisms related to strain competition. The step-change in replacement could have resulted from behavioral changes, potentially elicited by public health messaging or policies, that differentially affected Omicron.

## Teaser

The Omicron variant replaced the Delta variant in England despite the rate of replacement approximately halving in mid-December 2021.

## INTRODUCTION

The B.1.1.529 variant designated as Omicron was defined as a variant of concern (VOC) by the WHO on 26th of November 2021 (*1*). This variant caused international concern due to the exponential increase in infections observed in South Africa following the first detected case in Botswana on 11^th^ November 2021 (*2*). The variant spread globally, with the first sequenced case in the United Kingdom identified on 27^th^ November 2021 (*3*). Ascertaining whether Omicron transmission was going to be additive to or replacing the then-dominant Delta variant became an urgent priority for the UK’s public health response.

Early estimates of the growth rate of Omicron in South Africa found the doubling times of cases to be between 1 to 2 days (*4*). The Gauteng province of South Africa, which experienced a very high attack rate from the Delta variant, reported a doubling time of 2.0 to 3.3 days (*5*) for confirmed cases of the Omicron variant from 8^th^ November to 5^th^ December 2021. The variant subsequently spread across the African continent and on the 9^th^ of December it was reported that cases of the variant had increased by 93% within the past week (*6*). Omicron had been recorded in over 77 counties by the 14^th^ of December (*7*), with this a probable underestimate of its rapid geographic dispersion due to disparate levels of sequencing and screening. Omicron cases grew rapidly after it was introduced to Europe, with doubling times that were unprecedented in comparison to earlier waves of the Alpha and Delta variants. This was particularly pronounced in Denmark that reported doubling times close to 2 days (*8*), rates that were mirrored in England (*9*).

The Omicron variant has a higher likelihood of biological vaccine escape than Delta. The variant has 37 mutations on the receptor binding domain (RBD) (*10*). Consequently, in vitro studies noted lower neutralisation titers of convalescent and vaccine sera against the Omicron variant (*11*, *12*) and real-world data has demonstrated reduced vaccine effectiveness (*14*). Some of these mutations are believed to confer transmission advantages (*11*) and research will be required to understand the impact of the further mutations in this variant. Notably, polymerase chain reaction (PCR) antigen tests that measure cycle thresholds (CT) for three targets fail to amplify the *S* gene for Omicron. This contrasts with Delta samples, which amplify on all three.

Here, we present an analysis that was conducted on an ongoing, real-time basis in England after South Africa first alerted the international community to the emergence of Omicron. We analyzed TaqPath PCR tests that reported cycle threshold values for the *N*, *S*, and *ORF1ab* gene targets to identify both Omicron (through *S* gene target failure, SGTF) and Delta cases (positive all three genes). We developed a geographically stratified logistic growth model to examine the rate of replacement of the Delta variant by Omicron. Our analysis examined both regional patterns as well as dynamics at the “Lower Tier Local Authority” (LTLA) level. This model was used as part of the public health response to Omicron by forecasting the expected rate and timing of the replacement of the Delta variant. In mid-December, the expected percentage of SGTF cases began to diverge from typical logistic growth. We therefore allowed the model to estimate a “changepoint”, where the logistic growth rate of the proportion of tests compatible with Omicron slowed. In the model, this acted as a time-dependent binary switch that allowed us to quantify the extent to which the competitive advantage of Omicron decreased mid-December. Additionally, we adapted a version of the model that stratified the data by vaccine status and age group, to investigate trends within these specific subcategories. Using Generalised Additive models, we estimated the doubling and halving times for SGTF and triple positive cases during the Omicron wave. Frequentist results such as the GAM-derived growth rates are reported with 95% confidence intervals (CI), while outputs from the Bayesian model are reported as 95% credible intervals (CrI).

## RESULTS

### National Analysis

**Fig. 1.**
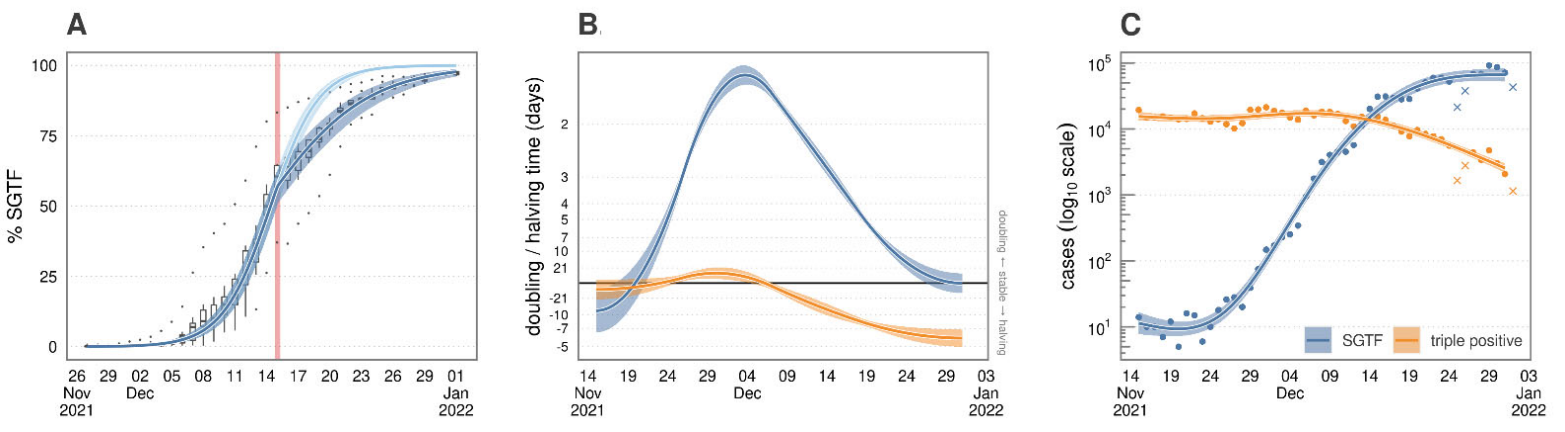
National trends of the relative and absolute growth of *S* gene target failure cases. (A) The fit of the logistic growth model to the proportion of *S* gene target failure (SGTF) cases through time. This model accounts for regional and lower tier local authority (LTLA) level heterogeneities in the replacement of the Delta variant with Omicron. The blue lines and ribbons denote the median and 95% credible intervals from the model fit; dark blue denotes the trend including the changepoint, the pale blue shows the expected path had the rate of replacement not changed. Red intervals show the estimated changepoint in the rate of replacement of triple positive with SGTF cases. Boxplots show the variation in the point estimates for the percentage of tests with SGTF in each English region. Note that lower and upper outliers are typically the North East and London, which respectively lagged and led the national average during the Omicron wave (see Fig. 2). (B) Doubling times of triple positive cases (blue) and SGTF cases (yellow) in England from the 15^th^ November 2021 to the 1^st^ of January 2022, as estimated by a GAM. The model fit is overlaid on case data in (C), with Christmas, Boxing Day, and New Year’s Day not included in model fitting as they were likely undercounts (denoted as crosses). Note, these are case rates within the subset of tests capable of detecting SGTF, not the total case rate for the England.

The model estimated that the national proportion of SGTF tests was 97.67% (95% CrI: 95.99, 98.63) by the 1^st^ of January 2022. The SGTF proportions and model predictions are shown in **Fig. 1A**; note that the regional heterogeneities (represented in the boxplots) are explicitly accounted for in the model framework. There was a changepoint – estimated as being between the 14^th^ and the 15^th^ of December – where the rate of replacement of triple positive by SGTF cases slowed. After the changepoint, the coefficient describing the rate of replacement was 53.56% (95% CrI: 45.38, 61.01) smaller than before, suggesting that the growth rate advantage of Omicron over Delta was approximately halved. In a separate analysis of the absolute growth rate of cases (**Fig. 1B** **-** **1C**), we estimated a peak doubling time of 1.56 days (95% CI: 1.49, 1.63) of SGTF cases on the 5^th^ of December. By contrast, triple-positive cases were approximately stable on the 5^th^, with confidence intervals including a 31.02 day doubling and 56.23 day halving. As England entered the Christmas holidays on the 24^th^ of December, triple-positive cases were halving every 5.82 days (95% CI: 5.11, 6.67), whereas SGTF cases were doubling every two weeks (14.55 day doubling, [95% CI: 21.35, 11.03]).

### Regional Analysis

**Fig. 2.**
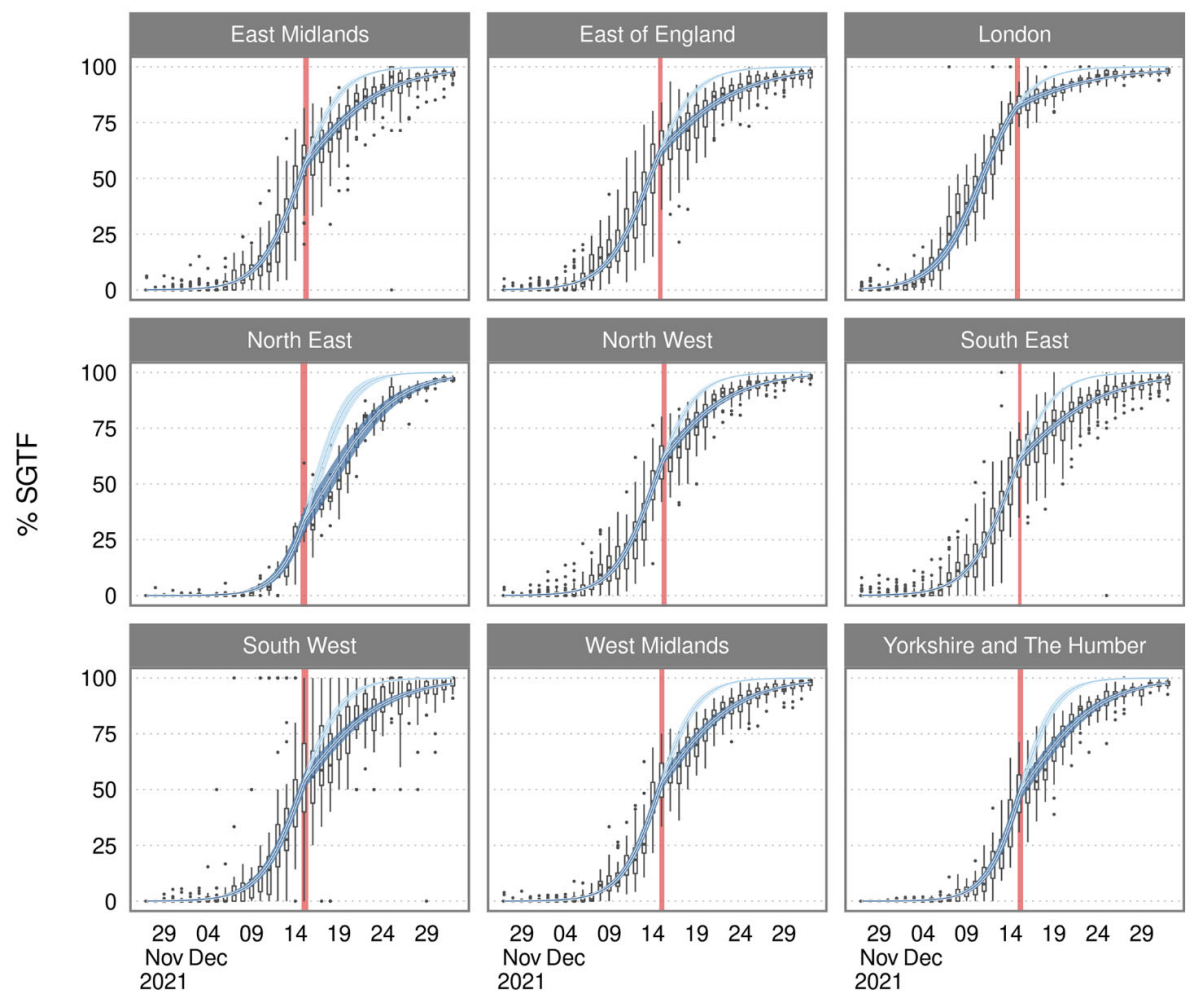
The proportion of *S* gene target failure and the growth rate model with credible intervals for each of the 9 regions of England. The blue line and ribbon denote the median and 95% credible intervals from the model fit; dark blue denotes the fit with the changepoint, the pale blue shows the path had the rate of replacement not changed. Red intervals show the model-estimated changepoint in the rate of replacement of triple positive with *S* gene target failure cases. Boxplots show the variation in the percentage of tests with SGTF across the lower tier local authorities (LTLAs) within each region.

Regional heterogeneity in the transmission of the Omicron variant is shown in **Fig. 2**. We allowed the model to estimate both region-specific and LTLA-specific changepoints in the rate of replacement of Delta by Omicron, with the latter hierarchically dependent parameters nested in the former. All regions had an estimated changepoint of between the 14^th^ and the 15^th^ of December. By the 1^st^ of January 2022, no region had an estimated percentage SGTF lower than 97%.

**Fig. 3.**
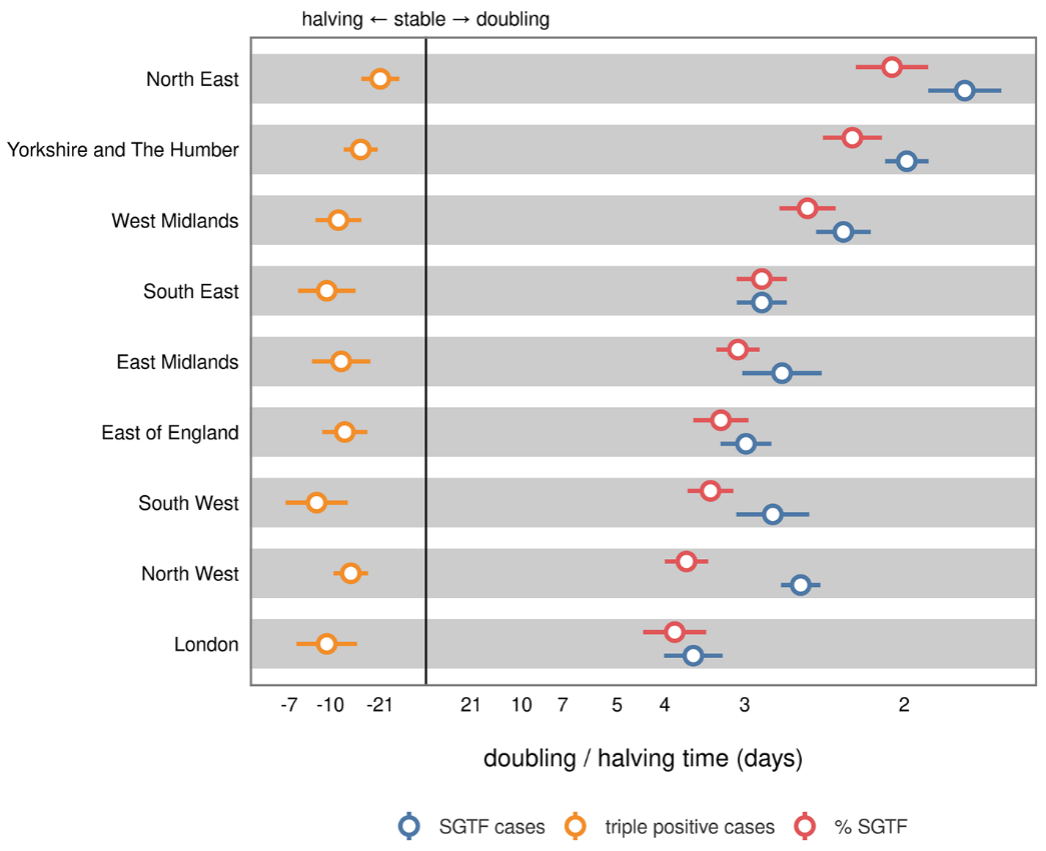
GAM-estimated doubling and halving times for *S* gene target failure, triple positive, and percentage of *S* gene target failure cases. Doubling and halving times were estimated using a GAM, as outlined in the methods. Trends are shown for the nine regions of England on the 14^th^ December, approximately when our logistic growth model estimated a step-change in the rate of replacement of triple positive with *S* gene target failure (SGTF).

London saw the earliest growth in SGTF, with 77.59% (95% CrI: 75.01, 80.05) of cases likely Omicron around the changepoint on the 14th of December. Early growth of SGTF in London was closely followed by neighboring regions in the East of England and the South East, reaching 53.65% (95% CrI: 50.76, 56.71) and 48.41% (95% CrI: 45.06, 51.99) respectively. The North East and Yorkshire lagged other regions considerably; both were well below 40% SGTF prior to the changepoint at 23.30% (95% CrI: 19.14, 27.60) and 35.12 (95% CrI: 30.83, 39.54), respectively. In **Fig. 3**, we show estimated growth rates in triple positive, SGTF and the percentage of SGTF across regions. We observed that the regions which experienced later SGTF growth – such as the North East and Yorkshire and the Humber – had the shortest absolute and relative doubling times at the changepoint. On the 1st of January, regions leading the epidemic such as London reported a 19.62-day (95% CI: 38.03, 7.80) halving time of SGTF cases whereas the North East was experiencing a rapid 4.92-day (95% CI: 7.22, 3.74) doubling.

**Fig. 4.**
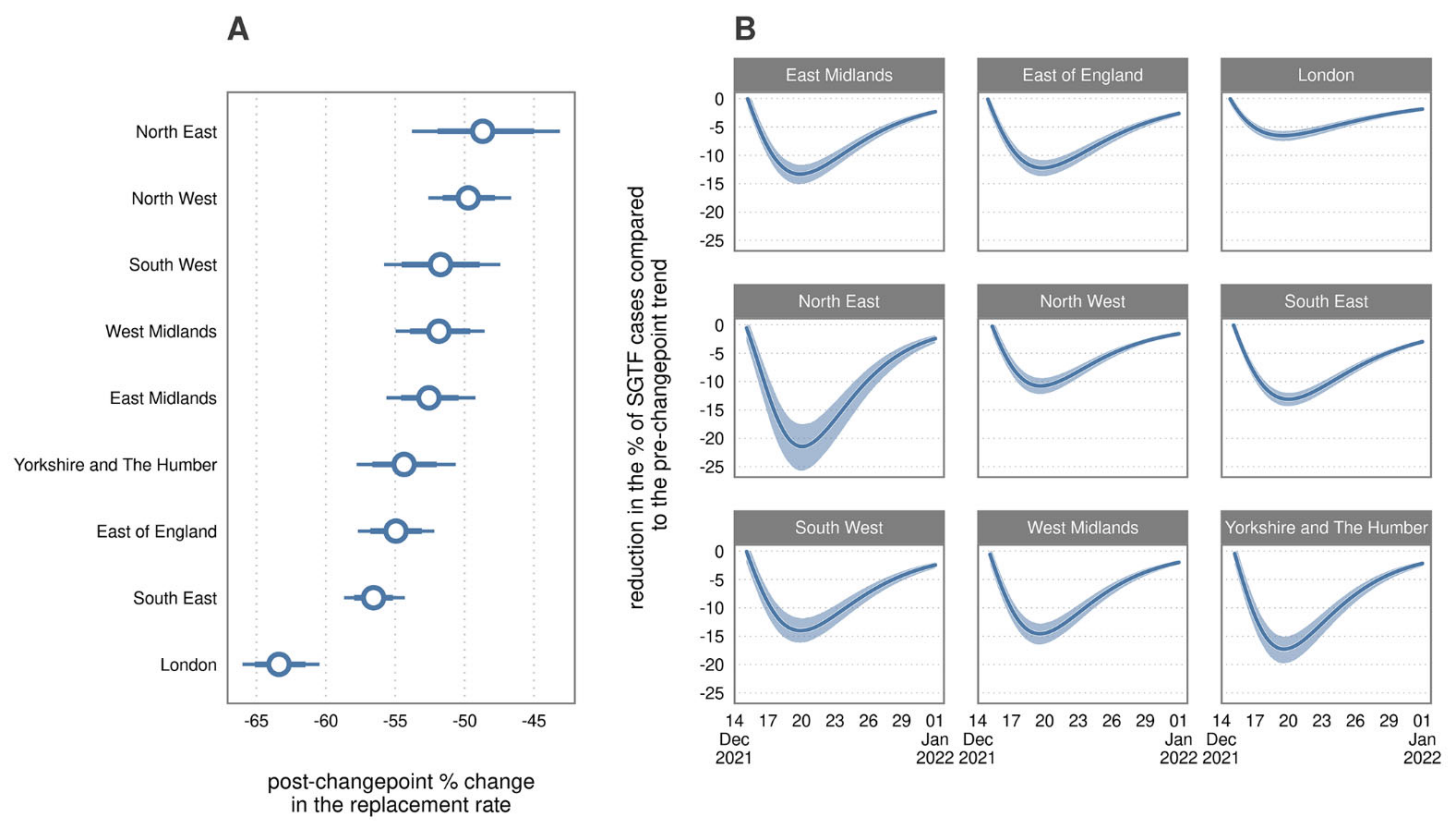
Illustration of the effect size associated with the regional changepoints in the rate of replacement. (A) Percentage reduction in the rate of replacement post changepoint (pone minus the ratio of the pre to post changepoint) with 80 and 95% CrIs. (B) Consequences of the rate reduction from (A) on percent *S* gene target failure (SGTF) after the changepoint with 95% CrIs.

**Fig. 4** illustrates the consequences of the changes in the rate of replacement for regional epidemics; **Fig. 4A** shows how the coefficient changed across the changepoint, whereas **Fig. 4B** shows the difference between the dynamics predicted with and without the changepoint. The effect size was greatest for London, where the rate of replacement of Delta by Omicron slowed by 63.37% (95% Crl: 60.57, 66.04). The North East, which experienced the latest growth in SGTF, had the largest realized effect size (**Fig. 4B**); by the 20^th^ of December, we observed 21.42% (95% Crl: 17.52, 25.42) lower SGTF than if the changepoint had not occurred. As the changepoint only reduced and did not reverse the fitness advantage of Omicron, anywhere with delayed growth in the near term would be expected to catch up eventually as Delta was replaced in the long term.

### Lower Tier Local Authority (LTLA)-level inference

**Fig. 5.**
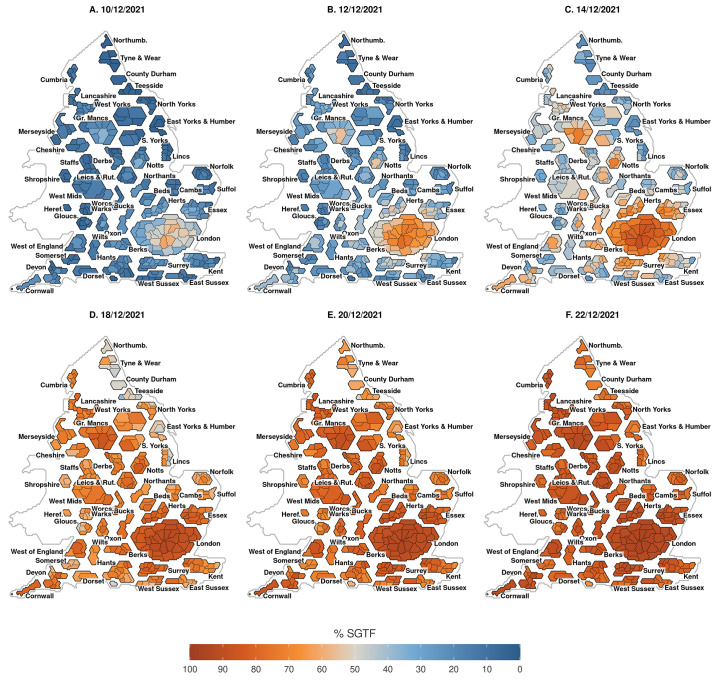
Progression of *S* gene target failure case growth at the LTLA level. Cartograms are presented using population weighted hexagons, displayed spatially based on approximate geographic location of the lower tier local authority (LTLA). Blue colors denote under 50%, grey 50% and red over 50% *S* gene target failure (SGTF) cases. Left to right are SGTF levels in two-day intervals from the 10^th^ to the 22^nd^ of December, modeled at the LTLA level.

In **Fig. 5**, we show the phases of SGTF growth between the 10^th^ and the 22^nd^ of December across the LTLAs in England; this date range was chosen to as it illustrates the most pronounced shift in SGTF cases. LTLAs in London, Greater Manchester, Nottinghamshire, Oxfordshire, and Northamptonshire experienced some of the earliest growth in SGTF. However, the initial outbreak was largely focused in and around London, with no LTLAs below 70% SGTF by early- to mid-December (**Figure S1**). LTLA-level model fits are shown in **Figures S1-S9** and illustrate intra-regional variation in Omicron epidemics.

### The role of vaccination status and age in the Omicron wave

**Fig. 6.**
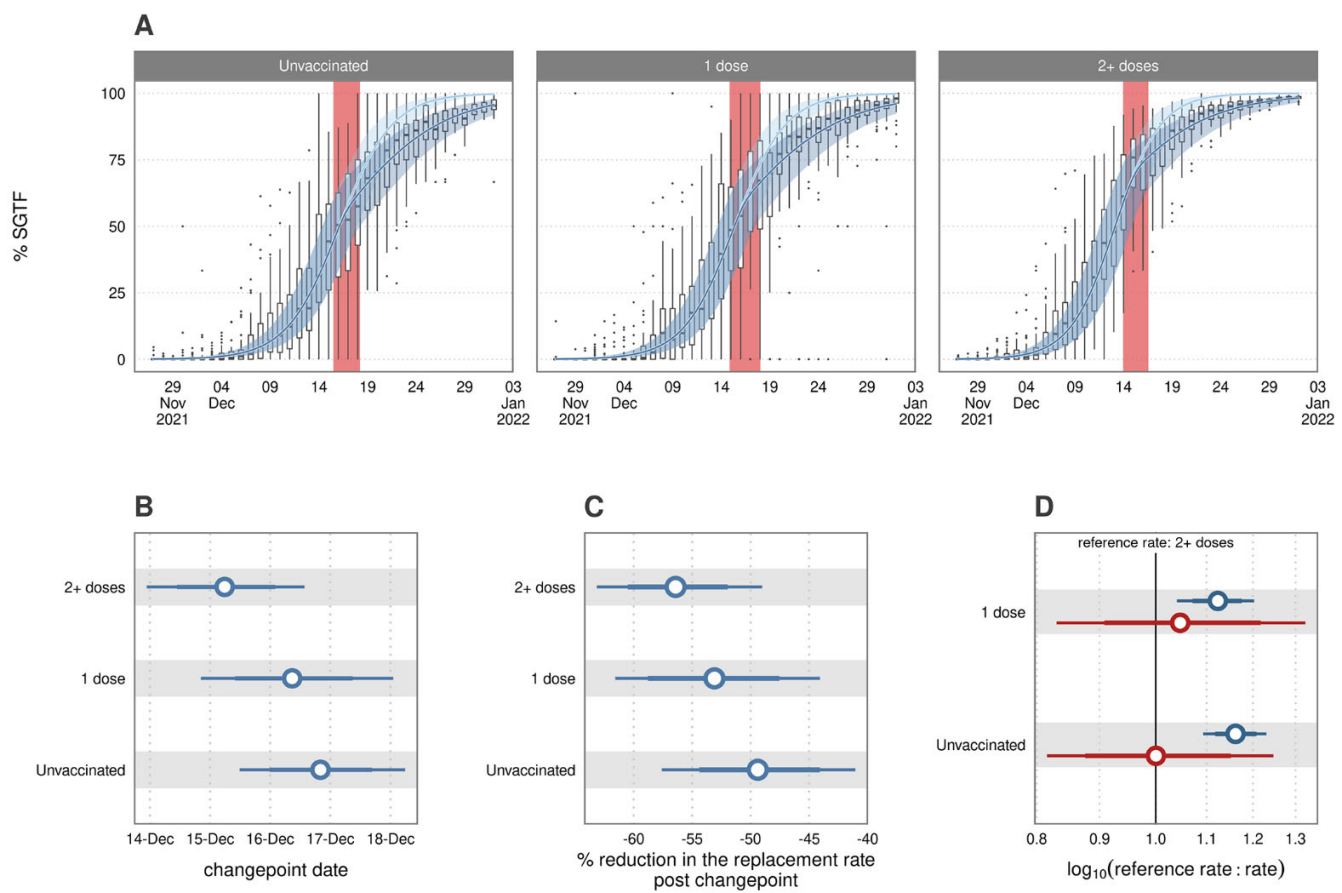
The effect of vaccine status on the replacement dynamics of Delta by Omicron. This model splits the data by vaccination status, and accounts for the effect of age and regional variation as nested random effects. (A) Vaccine status-model fit with 95% credible intervals. Boxplots show the variation in the point proportions in each age group and region; this variance is accounted for in the model inference. (B) Comparison of the changepoint split by vaccine status. (C) Summary of the percentage change in the rate of replacement after the changepoint for each vaccination group. (D) Log-ratio of the pre (blue) and post (red) changepoint rate of replacement in comparison to the corresponding reference rate. In this panel, the “reference rate” the will either be the pre or post changepoint rate for those 2 or more vaccine doses, while the “rate” is that written on the y-axis. A value of one indicates equal rates, greater than one indicates that the reference rate is larger than the comparison rate and less than one indicates the reference rate is smaller.

We tested a model stratified by vaccination status, where changepoints and replacement rates were estimated for those who were unvaccinated and those who were vaccinated with either one or two or more doses (there were insufficient numbers of breakthrough booster cases to include this as a separate factor level in the model). In **Fig. 6**, we present the rates of replacement split by vaccination status. Here, we account for the confounding factors of age and region in our model estimates. Omicron replaced Delta earlier (**Fig. 6A, B**) and 16.24% (95% CrI: 9.72, 23.41) faster (**Fig. 6C**) in those with two or more doses of the vaccine compared to the unvaccinated. Single dose and unvaccinated dynamics were broadly comparable. After the changepoint, the rate of replacement was most significantly reduced in vaccinated individuals (**Fig. 6C** **)** declining by 56.28% (95% CrI: 49.33, 63.11), such that the rate matched that of the other two groups (**Fig. 6D**). The changepoint date was estimated as slightly earlier for those with two or more doses, but the credible intervals include the central estimate of each subsequent group. Although the largest post changepoint reduction was estimated for those with two or more doses, there was again high uncertainty in the model estimates.

**Fig. 7.**
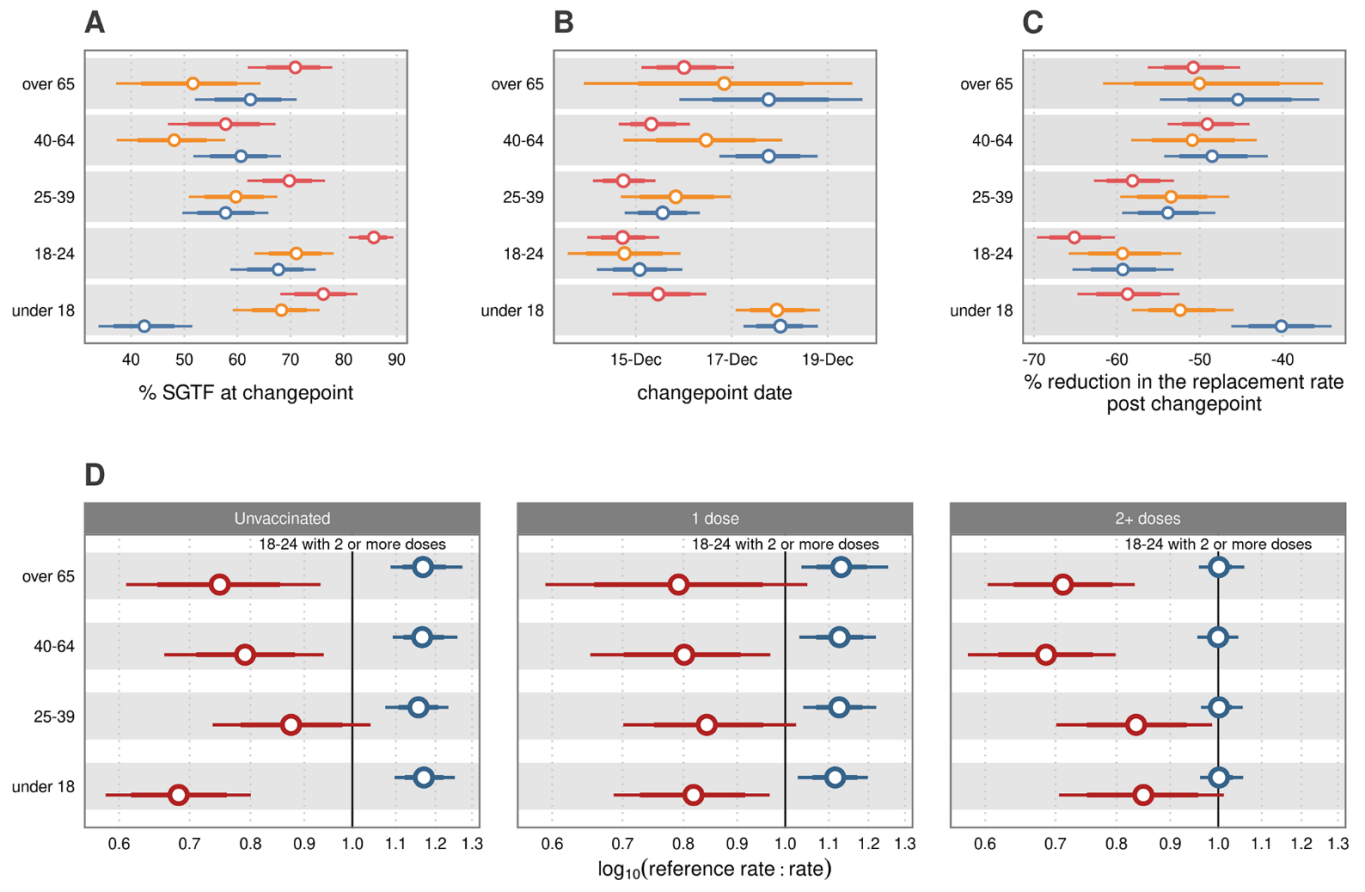
The interaction between vaccine status and age on the replacement dynamics of Delta by Omicron. (A) Age and vaccine status stratified percentage of *S* gene target failure (SGTF) cases at the changepoint (dates shown in (B)) with 80 and 95% credible intervals. The reduction in the rate of replacement is shown in (C). In (A-C), unvaccinated are shown in blue, single dose in yellow and two or more doses in red. (D) Log-ratio of the pre (blue) and post (red) changepoint rate of replacement in comparison to the corresponding reference group. Here, the “reference rate” is either the pre or post changepoint 18–24-year-olds with 2 or more doses with the rate given on the y-axis. The log ratio is the reference rate of replacement divided by the rate for the group written on the y-axis on the log_10_ scale. A value of one indicates equal rates, greater than one indicates that the reference rate is larger than the comparison rate and less than one indicates the reference rate is smaller.

In all regions, 18–24-year-olds who had been double vaccinated or boosted comprised the leading wave of Omicron infections up until the changepoint; on the 15^th^ of December 85.67% (95% CrI: 81.04, 89.33) of cases in this group were probable Omicron (**Fig. 7A**). The timing of the age-specific changepoints is compatible with the vaccinated 18-24- and 25–39-year-old age groups driving the replacement in the older age groups (**Fig. 7B**). This was evidenced by the ordering of the age-dependent changepoints, with the earliest change occurring in these two age groups, followed by the over 40s. If within age-group transmission was independent, it would be likely that the changepoint would have been consistent across all ages. The unvaccinated under 18s lagged in the progression of SGTF (**Fig. 7A**), following independent dynamics in terms of changepoints and replacement dynamics. **Fig. 7D** provides evidence that the pre-changepoint fitness advantage of Omicron in those with more than two doses was agnostic to age. The fit of the vaccine status model is given in **Figure S10** and regional trends for each age group and vaccination status in **Figure S11**.

## DISCUSSION

Our analysis documents the almost complete replacement of Delta by Omicron in England entering the New Year, despite the first sequenced case being identified just over a month before on the 27^th^ of November 2021. The growth in the proportion of SGTF tests was led by London and South East. However, by approximately the 15^th^ of December, we had observed a slowdown in the rate of replacement. At this point, the North East, West Midlands, South West and Yorkshire were yet to reach 50% replacement, and we have shown that this likely prolonged the period of growth in absolute numbers of SGTF cases as Omicron’s fitness advantage was yet to be fully realized. The lower observed proportions of SGTF in the North East, West Midlands, South West, and East Midlands may also be impacted by a smaller fraction of tests sent to laboratories that report PCR gene targets in these regions (*12*) which can bias the analyses.

Strain competition theory predicts that trade-offs between cross-protective immune responses, immune evasion (including from vaccination) and transmission advantages that can facilitate coexistence of variants (*13*, *14*). There was therefore a realistic possibility that we would have suffered parallel winter epidemics of Omicron and Delta in the United Kingdom. The antigenic novelty of Omicron may have granted an advantage in vaccinated and previously infected individuals, with Delta benefitting from a transmission advantage among the immunologically naïve. However, few individuals in the United Kingdom are entirely naïve to SARS-CoV-2 due to widespread vaccine coverage and considerable numbers of infections. The rate of Delta cases prior to Omicron’s arrival would have generated not inconsiderable Delta-specific population immunity. This makes it unlikely that Delta could maintain cycles of transmission, and there is currently no evidence to suggest Delta is more competitive in an immunologically naïve population. The changepoints estimated by the model are indicative of a step change in the competitive advantage of Omicron over Delta. Although Omicron retains its advantage after the changepoint, it is considerably – 53.56% (95% CrI: 45.38, 61.01) – smaller than before. We found that the pre-changepoint rate of replacement was 16.24% (95% CrI: 9.72, 23.41) faster in those with two or more doses of the vaccine. This was not necessarily due to a faster rate of spread in absolute terms for this group, rather that the fitness advantage over Delta was greatest, causing replacement to occur more rapidly. This is compatible with vaccine evasion by Omicron and points to this variants ability to exploit this population more than Delta was able to. The significant evasion of anti-infection immunity was unaccompanied by an equivalent reduction in anti-disease immunity, which remains comparatively robust. Reductions in Omicron’s severity are potentially due to it replicating more effectively higher in the respiratory tract (*15*).

Interpreting the post changepoint dynamics is challenging, as many mechanistic explanations are credible and will be confounded by vaccine, age, and spatial heterogeneities. It is outside the scope of this correlative analysis to allocate one or more mechanistic explanations with certainty. We can comment that a component of Omicron’s competitive advantage is likely due to vaccine escape (evidenced by the pre-changepoint growth rate advantage in those with two or more doses) and is supported by real world vaccine effectiveness (*16*). The robustness of this estimated effect to age suggests that there is a mechanistic basis to this advantage. What is unclear is why the benefit of vaccine escape reduced to a slightly greater extent than the unvaccinated or single doses after the changepoint; the post-changepoint rates of replacement are broadly comparable across vaccine groups. It is possible that fully vaccinated individuals may have temporally correlated behavioral responses that would mitigate the competitive advantage of Omicron. We feel confident in suggesting that it is unlikely that a distinct, binary changepoint with a significant effect size is associated with a change in vaccine induced immunity (such as the waning of infection-blocking immunity from previous doses or increased population immunity from the rollout of booster doses). Notably, an important component of immune escape is infection-acquired versus vaccine-acquired immunity; among some groups, Delta-specific immunity may not provide sterilising immunity against Omicron, but likely will do so against Delta, at least in the near term. The feedback of the Delta-induced immunity will act against Delta transmission to the benefit of Omicron. Among some groups, the attack rate of Delta may have been substantial, and is likely to overlap with those who led the Omicron wave.

Dynamics in the under 18s are difficult to interpret, particularly in relation to vaccination. This group is diverse in terms of vaccination policy (for example targeting vaccination toward clinically at-risk groups). Moreover, there are diverse within-group contact patterns; for instance, those 16 plus may have contact patterns closer to an 18-year-old. It is notable that the unvaccinated under 18s had a relatively late changepoint (around the 18^th^ of December) at which point only around 42% of cases were compatible with Omicron, by far the lowest group in England at that time. After the changepoint, unvaccinated under 18s had the smallest reduction in the rate of replacement.

Preceding the changepoint in the rate of replacement, the UK government enacted the “Plan B” policy, a suite of measures aiming to curtail transmission (*17*). This included mandatory face covering on public transport and most indoor venues from the 10^th^ of December, an advisory work from home notice from the 13^th^ and a requirement for proof of a negative test or vaccination through the “NHS Covid Pass” for large gatherings from the 15^th^. Moreover, media coverage, public health messaging, and a 10-day isolation after a positive test (*18*) may have precipitated behavioral change that reduced contacts ahead of the upcoming Christmas period. Mobility – to the workplace and hospitality – reduced over this period (*19*). The shorter generation time of Omicron may mean that behavioral changes may have a disproportionate impact on this variant compared to Delta (*20*, *21*). It would be expected that a variant with a shorter generation time would be more responsive to the observed changing patterns of behavior since transmission events would be prevented earlier relative to a variant with a longer generation time. Crucially, correlative analyses such as the one we present here cannot ascribe specific causational impacts of behavioral and policy changes on replacement rates.

The Omicron variant was detected through SGTF (*22*) due to an *S* gene deletion, which affects tests that use the TaqPath COVID-19 CE-IVD RT-PCR Kit (*23*). This deletion allows for more timely surveillance of this novel variant through the routine case data (the lead time on sequencing is between 10 and 14 days). Low viral load Delta can also produce SGTF and analysis therefore must be conducted on low CT (< 30) cases. The Alpha variant also carried the 69-70del mutation and low CT SGTF may still be the Delta variant, so analysis must adjust for a constant background rate of SGTF in the testing data. We observed considerable divergence from the background rate of SGTF that could be attributed to the Alpha or Delta variants, and this therefore illustrates the strength of the SGTF signal to identify the Omicron variant. Our exclusion criteria for tests ensures high sensitivity and specificity of SGTF to Omicron across the period we studied.

Not all laboratories report PCR gene targets and this data has a geographic bias. One study found that the South of England, particularly the South West, East Midlands, and East of England, have the lowest laboratory reporting of PCR gene targets (*12*). The North West benefits from the highest reporting rate, where 90% of all PCR tests reported gene targets. There was an increased use of TaqPath laboratories since the 15^th^ November to detect the Omicron variant, which has improved the geographic reporting coverage. More generally, test seeking behavior can be confounded by various demographic variables. For instance, the older (65+) age group was poorly sampled as engagement with Pillar 2 testing is lower (these cases are often identified on admission to hospital). Moreover, the financial and practical costs of self-isolation are often higher for those who cannot work remotely (which is often also correlated with socio-economic status), changing test-seeking behavior. We have included only low-CT symptomatic cases to minimise any biases from the testing data, but no criteria can perfectly compensate for all Pillar 2 testing confounders.

In the models, we allowed for the breakpoint to be estimated within each hierarchically nested variable. These breakpoints will represent the best improvement to the model fit across the time series with respect to a particular level of a geographic or demographic variable. This does not preclude the existence of other changepoints in the rate of replacement, but we are confident that these modeled breakpoints represent the most substantial change in the rate in the month of December.

The replacement of Alpha by Delta coincided with non-pharmaceutical interventions (NPI) at the start of 2021, which may have contributed to Alpha cases declining from the 7^th^ January 2021 (*12*). We observed exponential growth in Delta around step 1a of lockdown easing (*24*); London, Yorkshire and the Humber, North East, and the West Midlands experienced the fastest growth in Delta. A similar pattern was observed for Omicron, despite differential importation patterns and seeding into different communities. London appears to have experienced the earliest growth, with Yorkshire and the Humber and the North East lagging behind. We also observed that LTLAs in the North West of England experienced early exponential growth in Omicron, which were also observed in growth patterns of Delta.

Soon after its introduction, determining the severity of Omicron cases from reinfections or vaccine breakthrough was essential. However, at that time, the epidemic was predominantly in the 18-39 age group where clinical outcomes were mild. Omicron is capable of substantial immune evasion, however additional “booster” doses of existing vaccines confer higher neutralising antibody titers against Omicron, albeit to a lesser extent than for other variants (*25*–*27*). Nonetheless, the interplay between immune responses, evasion, and severe disease is complex, particularly in the vulnerable. Caution must be exercised when conducting inter-country comparisons of severity, as differences in demography, exposure, and vaccine coverage/regimes confound data. Unprecedented rates of Omicron cases were associated with a concomitant rise in hospitalisations, but at a considerably lower rate than under Delta. This was due in large part to booster rollouts to the most vulnerable in the UK, existing anti-disease immunity (from infection and vaccination), and reduced severity of Omicron infection.

Our analysis highlights the utility of using gene target proxies in monitoring an emerging variant. However, our exercise also demonstrates the challenges involved in estimating the rate of replacement in real time. In the absence of a well-parametrised mechanistic model which captures the specific cause of the fitness advantage, we were unable to anticipate impacts of externalities on the rate of replacement. Mechanistic models rely on adequate parameterisation and credible initial conditions. In the case of Omicron entering the UK, the variant invaded a system of high vaccine coverage (with various states of waning and booster delivery) and a rolling rate of Delta infections (eliciting strong immunity against this specific variant); this would need to be accurately represented in a mechanistic model and would require several key assumptions. The extent to which the fitness advantage of Omicron comes from faster transmission or immune evasion would be key parameters that were not quantified ahead of predictive modelling efforts. Moreover, to predict the change in fitness advantage, models would need to account for host behavior (if that was indeed the cause of the changepoint). This leaves a large region of credible parameter space to explore. Post-hoc analyses are therefore more likely to identify the specific mechanistic explanations of this takeover as opposed to real-time monitoring efforts. Certainly, real-time monitoring can – as it did in our case – quickly identify that reality has deviated from our modeled expectations, which enables a nimble response to changing circumstances.

Mass testing datasets contain numerous confounders that cannot all be accounted for in the model, some of which we have documented above. For this reason, we minimised our interpretation of absolute case growth, focusing instead on the representation of SGTF in relation to all tests. This is because we anticipated that changes in the representation of Omicron in the sample of tests within locations and demographics would be comparatively robust to testing confounders, unlike absolute numbers. This precludes us from commenting on the effects of the breakpoint on the overall burden of cases, or specific vaccine- and age-related differences in the force of infection during the Omicron wave. Moreover, the effect of variables such as vaccination status must be parsimoniously represented in the model to maximise the power of our inference. For instance, we do not account for the timing of vaccine doses, either in relation to the building of the immune response or subsequent waning. Last, geographies such as regions are ultimately artificial groups of local authorities that do not necessarily reflect the epidemiology of constituent LTLAs, particularly when on a border. We have therefore been careful and specific in limiting the causative interpretation of our models.

By the 1^st^ of January 2022 the Omicron variant had become dominant in England, almost entirely replacing the Delta variant. We found considerable geographic heterogeneity in the timing of Omicron epidemics with similar patterns of regional growth observed for the emergence of Delta. Omicron replaced Delta more quickly in the vaccinated before the changepoint, strongly suggesting that vaccine escape was a key driver of the fitness advantage. We observed a step-change in the rate of replacement of Delta in mid-December when the competitive advantage of Omicron approximately halved. The synchrony of the changepoints across regions – which were at various stages of Omicron epidemics – suggest that Omicron could have suffered a reduction in relative fitness concurrent to changes in host behavior.

## MATERIALS AND METHODS

### Study Design

This study aimed to determine how the Omicron variant spread in England after its introduction in November 2021. Specifically, we considered how the frequency of the novel variant changed in relation to the then dominant Delta variant, accounting for geographic and demographic confounders. We analyzed polymerase chain reaction (PCR) testing data from Second Generation Surveillance System (SGSS) dataset, which is the central database for community, or ‘Pillar 2’, tests in the United Kingdom. PCR tests were provided free of charge across the study period, but data are confounded by test seeking behavior that will vary by demographics (socio-economic, age etc). These tests provided a proxy for Omicron and Delta cases, which we outline below. We analyzed data from the 15^th^ of November 2021 up to 1^st^ January 2022; after this point, the emergence of the Omicron variant BA.2 would have complicated analysis and interpretation. Only data from England were included, as complete data was unavailable for the devolved administrations of the UK (Northern Ireland, Scotland and Wales).

We extracted information on PCR results from the laboratories that use TaqPath RT PCR kits (*23*) in the United Kingdom: Milton Keynes, Newcastle, Glasgow, and Alderley Park Lighthouse Laboratories. Other private sector labs with reliable gene target data were also included. Only symptomatic cases were analyzed to keep test seeking behavior as consistent as possible. Tests with SGTF (*S* gene negative) and those that were positive for all 3 genes (*N*, *S* and *ORFab* positive) were used as a proxy from the Omicron variant and Delta variant, respectively. Not all regions of England have equal coverage of TaqPath tests, meaning the gene-target based estimates of Omicron prevalence will be more uncertain in some locations. The outlined combination of inclusion criteria yielded a total of 1,542,211 positive PCR tests for the analysis; 955,455 of these were SGTF and therefore compatible with being Omicron BA.1.

There had been a background rate of SGTF in the testing data in England prior to Omicron. Since August 2021 this background rate had been increasing and was predominantly attributable to low viral load cases of Delta where the *S* gene fails to amplify due to degraded RNA in the sample. To increase specificity, we only modeled tests with the CT values < 30 for both the *N* and *ORF1ab* genes, and an *S* gene CT value <30 (triple positive) or a failure to amplify (SGTF). To test the sensitivity and specificity of this definition of SGTF for Omicron BA.1, we matched test’s reporting cycle threshold values for all three targets with confirmed sequenced cases. Under our definition, SGTF was 99.26% (CI: 99.23, 99.30) sensitive and 99.92% (CI: 99.91, 99.93) specific to Omicron BA.1. As of the end date of our analysis, only 13 cases of Omicron BA.2 that could be matched to gene target data that had been sequenced in England (all triple positive). This makes it unlikely that the patterns of replacement we describe were confounded by the emergence of BA.2.

### Instantaneous Growth Rate

We determined the time varying instantaneous growth rates for triple positive and SGTF cases using Generalised Additive Models (GAM) with a negative binomial error structure and canonical log-link (*12*, *28*). We fit cubic regression splines and tuned the optimal number of knots by assessing the model fit to case rates (*29*). A random effect on the day of week accounted for cycles in positive test numbers that typically peak mid-week. The model assumed the number of cases y(t) is proportional to $\exp\left(s\left(t\right)\right)$ for some smoother. We estimated the instantaneous growth rate as a time derivative of the smoother $r_{s}=\dot{s}\left(\text{t}\right)$ , and the instantaneous doubling time is calculated as $t_{D}=\log\left(2\right)/\dot{s}$ . The asymptotic confidence intervals (CIs) on $r_{s}$ are indicative of the uncertainty on $t_{D}$ .

### Bayesian Hierarchical Logistic Growth Model

We modeled the logistic growth of the proportion of tests with SGTF through time. All regions and Lower Tier Local Authorities (LTLAs) are fit using a hierarchical model where the national model fit is an average of all regional and LTLA fits. The model structure allowed for a changepoint in the rate of replacement, where the rate could increase or decrease beyond a certain point in time. We model the proportion of tests with SGTF in each LTLA (out of the total *L*) nested within region *r* (out of the total *R*) using the equations:

$$logit\left(p^{r,l}\right)=\left\{\begin{array}{c} T<\tau^{r,l},\;\;\alpha^{r,l}+\beta_{T<\tau^{r.l}}^{r,l}\times \left(T-\tau^{r,l}\right)\\ T\geq \tau^{r,l},\;\;\alpha^{r,l}+\beta_{T\geq \tau^{r,l}}^{r,l}\times \left(T-\tau^{r,l}\right) \end{array}\right.$$

Here, $p^{l,r}$ gives the estimated proportion of tests with SGTF. The parameter $\alpha^{r,l}$ . is the intercept and the $\beta^{r,l}$ parameters are the rate of increase toward replacement of Delta by Omicron, pre or post replacement. Another parameter, $\tau^{r,l}$ , determines the timing of a changepoint in the rate of replacement, which is region- and LTLA-specific. The coefficient $\beta_{\text{T}<\tau^{r,l}}^{r,l}$ is the rate of replacement prior to the changepoint and $\beta_{\text{T}\geq \tau^{r,l}}^{r,l}\;$ is the rate on and after the changepoint.

All parameters for regional and LTLA level fits were drawn from hierarchical distributions. The regional trajectories were taken from distributions where the intercept ( α ), rate of replacement and $\beta_{T\geq \tau }$ ) and the timing of the changepoint ( τ .) are the national averages and ${\text{σ}}_{{\text{α}}^{R}}$ , ${\text{σ}}_{{\text{β}}_{T<\text{τ}}^{R}}$ , ${\text{σ}}_{{\text{β}}_{T\geq \text{τ}}^{R}}$ and ${\text{σ}}_{{\text{τ}}^{R}}$ are the standard deviations for regional draws from the national distribution:


$$\alpha^{r}\sim N\left(\alpha ,\sigma_{\alpha^{R}}\right)$$

$$\beta_{T<\tau }^{r}∼N\left(\beta_{T<\tau },\sigma_{\beta_{T<\tau }^{R}}\right)$$

$$\beta_{T\geq \tau }^{r}∼N\left(\beta_{T\geq \tau },\sigma_{\beta_{T\geq \tau }^{R}}\right)$$.

$$\tau^{r}∼N\left(\tau ,\sigma_{\tau^{R}}\right)$$

Local authority level parameters were drawn from a second set of distributions where the mean is now the regional average (each LTLA is nested within a partilar region):


$$\alpha^{l}∼N\left(\alpha^{r},\sigma_{\alpha^{L}}\right)$$

$$\beta_{T<\tau }^{l}∼N\left(\beta_{T<\tau }^{r},\sigma_{\beta_{T<\tau }^{L}}\right)$$

$$\beta_{T\geq \tau }^{l}∼N\left(\beta_{T\geq \tau }^{r},\sigma_{\beta_{T\geq \tau }^{L}}\right)$$

$$\tau^{l}∼N\left(\tau^{r},\sigma_{\tau^{L}}\right)$$

We assumed that the within-region variance (that is, LTLA-level variance) was the same for each parameter. This model was fit with a binomial error structure, where the number of SGTF cases (S) is predicted by the probability given by the changepoint model and the total number of tests (*N*):


$$S^{r,l}∼Binomial\left(p^{r,l},N^{r,l}\right)$$

We were also interested in the effect of vaccine status and age in the replacement of Delta by Omicron. However, there was insufficient data at the LTLA level to disaggregate the full model by these two variables. Instead, we modified the above changepoint model such that vaccine groups were included as a fixed effect, with unvaccinated, single and two or more dose categories. We had to amalgamate those with two or more doses into one class as breakthrough infections of boosted individuals were comparatively rare. Age was split into 5 groups; under 18, 18-24, 25-39, 40-64 and over 65, and was included as a random effect nested within vaccine status. Spatial heterogeneity was modeled with a regional random effect, nested withing the fixed effect of vaccination status and random effect of age. This ensured that all confounding interactions of age and spatial heterogeneity were considered when modelling differences between vaccinated groups.

As before, we allowed for a changepoint in the rate of replacement. We model the proportion of tests with SGTF in each region *r* (out of the total *R*), nested within each age *a* (out of the total *A*) nested within each vaccine status *v* (out of the total *V*). Our rationale is that vaccination status is the focal and potentially mechanistic source of fitness advantages for Omicron, with age-specific trends and regional differences generally a matter of timing. The model is therefore:

$$\text{logit}\left(p^{v,a,r}\right)=\left\{\begin{array}{c} \text{T}<\tau^{v,a,r},{\text{ α}}^{v,a,r}+\beta_{\text{T}<\tau^{a,r}}^{v,a,r}\times \left(T-\tau^{v,a,r}\right)\\ \text{T}\geq \tau^{v,a,r},{\text{ α}}^{v,a,r}+\beta_{\text{T}\geq \tau^{v,a,r}}^{v,a,r}\times \left(T-\tau^{v,a,r}\right) \end{array}\right.$$

Here, $p^{v,a,r}$ gives the proportion of tests with SGTF in vaccine group *v*, age group *a* and region *r*. The parameter $\alpha^{v,a,r}$ is the intercept and $\beta^{v,a,r}$ is the rate of increase toward replacement of Delta by Omicron. Another parameter, $\tau^{v,a,r}$ , determines the timing of a breakpoint in the rate of replacement, which is vaccine status, age- and region-specific; $\beta_{\text{T}<\tau^{a,r}}^{v,a,r}$ is the rate prior to the breakpoint, $\beta_{\text{T}\geq \tau^{v,a,r}}^{v,a,r}\;$ is the rate on and after the breakpoint.

Each vaccine group parameter (intercept, replacement rates, and changepoint date) were treated as a fixed effect. All parameters for age and region level fits were drawn from hierarchical distributions. The age trajectories were taken from distributions where the intercept ( $\alpha^{v}$ ), rate of replacement ( $\beta^{v}$ ), and the timing of the breakpoint ( $\tau^{v}$ ) are the vaccine group averages and $\sigma_{\alpha^{V}}$ , $\sigma_{\beta_{T<\tau }^{V}}$ , $\sigma_{\beta_{T\geq \tau }^{V}}$ and ${\text{σ}}_{{\text{τ}}^{V}}$ are the standard deviations for the draws for each age category from the global distribution:


$$logit\left(\alpha^{a}\right)∼N\left(\alpha^{v},\sigma_{\alpha^{V}}\right)$$

$$log\left(\beta_{T<\tau }^{a}\right)∼N\left(\beta^{v},\sigma_{\beta_{T<\tau }^{V}}\right)$$

$$log\left(\beta_{T\geq \tau }^{a}\right)∼N\left(\beta^{v},\sigma_{\beta_{T\geq \tau }^{V}}\right)$$

$$log\left(\tau^{a}\right)∼N\left(\tau^{v},\sigma_{\tau^{V}}\right).$$

Regional corrections to the age-dependent trends were drawn from a second set of distributions where the mean is now the age-group (nested within vaccine status) average:

$$logit\left(\alpha^{r}\right)∼N\left(\alpha^{v,a},\sigma_{\alpha^{A}}\right)$$

$$log\left(\beta_{T<\tau }^{r}\right)∼N\left(\beta^{v,a},\sigma_{\beta_{T<\tau }^{A}}\right)$$

$$log\left(\beta_{T\geq \tau }^{r}\right)∼N\left(\beta^{v,a},\sigma_{\beta_{T\geq \tau }^{A}}\right)$$

$$log\left(\tau^{r}\right)∼N\left(\tau^{v,a},\sigma_{\tau^{A}}\right)$$

We assumed that the within-age group variances were the same for each parameter, denoted ${\text{σ}}_{{\text{α}}^{A}}$ , $\sigma_{\beta_{T<\tau }^{A}}$ , $\sigma_{\beta_{T\geq \tau }^{A}}$ and $\sigma_{\tau^{A}}$ . This model was fit with a binomial error structure, where the number of SGTF cases (S) is predicted by the probability and the total number of tests (N):


$$S^{v,a,r}∼Binomial\left(p^{v,a,r},\;N^{v,a,r}\right)$$

Logistic growth models were fit in the Bayesian modelling program “stan” version 2.21.0 (*30*) using the statistical programming language “R” version 4.0.2 (*31*). GAMs were fit using the package “mgvc” version 1.8-31 (*29*), also interfaced through R. Maps were produced using geographical files from the House of Commons Library under the Open Parliament License v3.0. Frequentist results such as the GAM-derived growth rates are reported with 95% confidence intervals (CI), whereas outputs from the Bayesian model are reported as 95% credible intervals (CrI). Priors are given in table S1.

## Supplementary Materials

### Other Supplementary Material for this manuscript includes the following:

MDAR Reproducibility Checklist
