# Supplementary material for: The rapid replacement of the Delta variant by Omicron (B.1.1.529) in England
Source: Sci Transl Med. 2022 May 3:eabo5395. doi: 10.1126/scitranslmed.abo5395 (PMC9097877; doi:10.1126/scitranslmed.abo5395)
Supplement: Supplementary file 1 — Figs. S1 to S11 Table S1 [file scitranslmed.abo5395_sm.pdf]

Science Translational Medicine
AAAS

Supplementary Materials for

The rapid replacement of the Delta variant by Omicron (B.1.1.529) in England

Robert S. Paton, Christopher E. Overton, Thomas Ward*

*Corresponding author: tom.ward@ukhsa.gov.uk

DOI: 10.1126/scitranslmed.abo5395

The PDF file includes:

Figs. S1 to S11
Table S1

Other Supplementary Material for this manuscript includes the following:

MDAR Reproducibility Checklist

Supplementary Material for:

THE RAPID REPLACEMENT OF THE DELTA VARIANT BY OMICRON (B.1.1.529) IN ENGLAND

Robert S. Paton, Christopher E. Overton & Thomas Ward*

Data Science and Analytics, UK Health Security Agency, Nobel House, London, UK, SW1P 3JR

*Corresponding author: tom.ward@ukhsa.gov.uk

Supplementary Figures

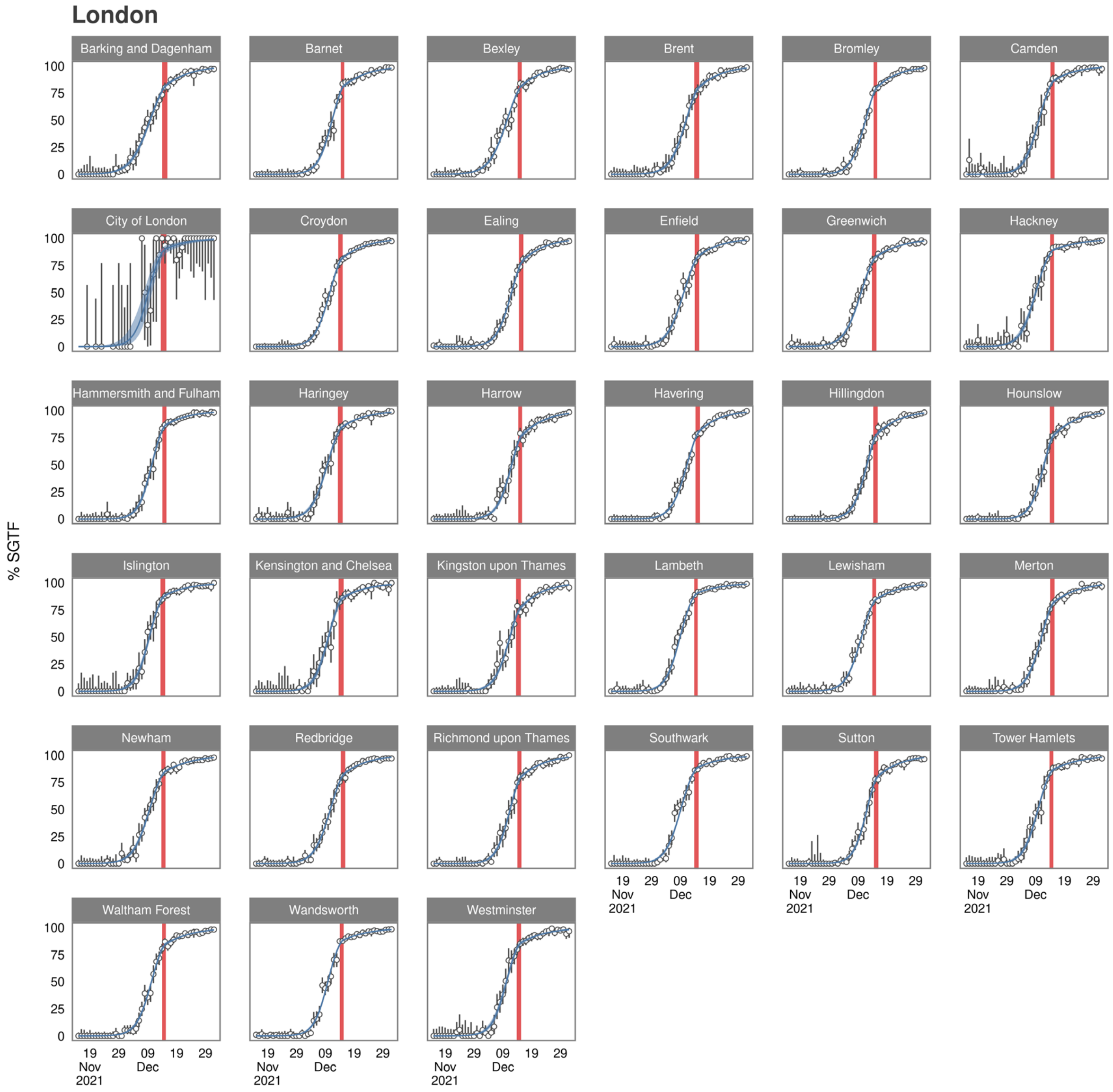

Figure S1: Model fit for London LTLAs.

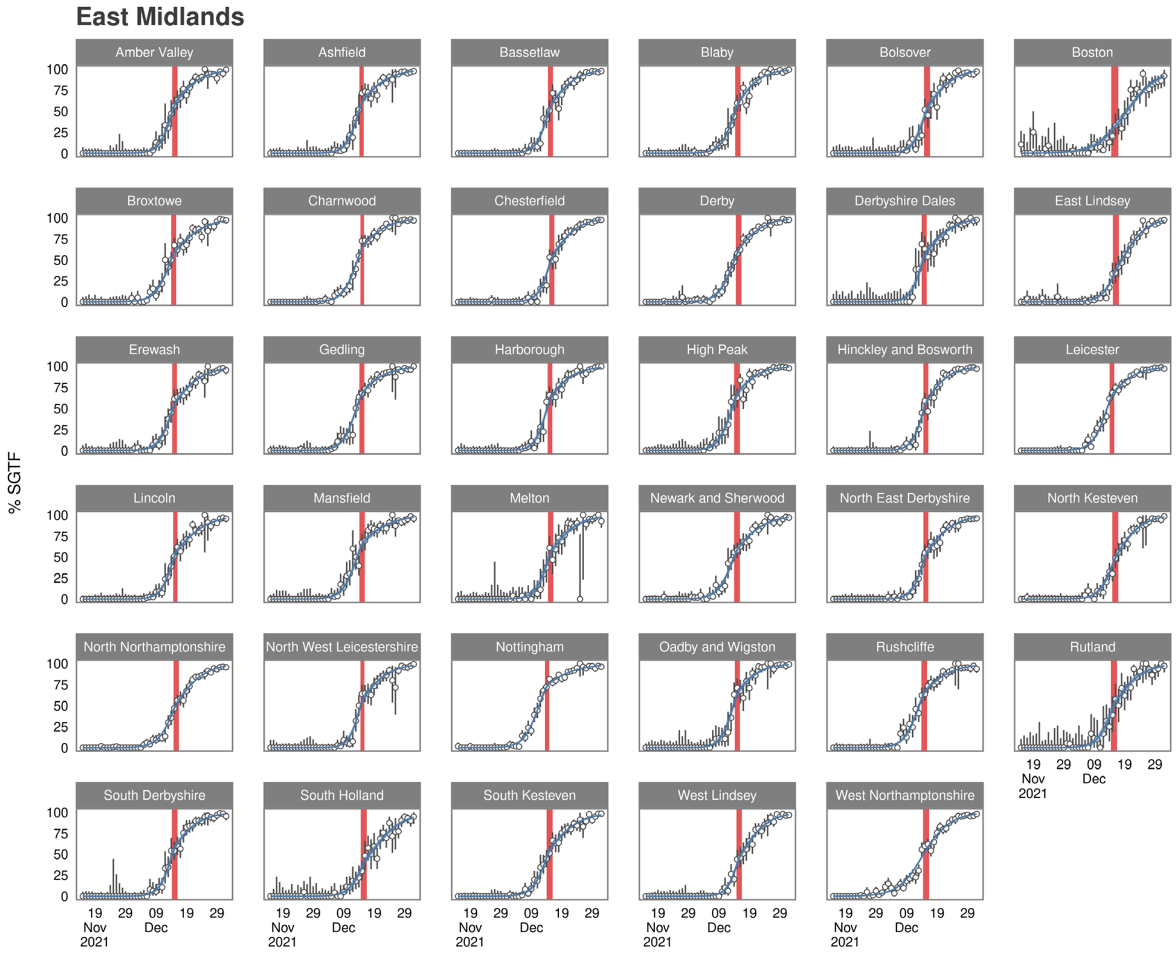

Figure S2: Model fit for LTLAs in the East Midlands.

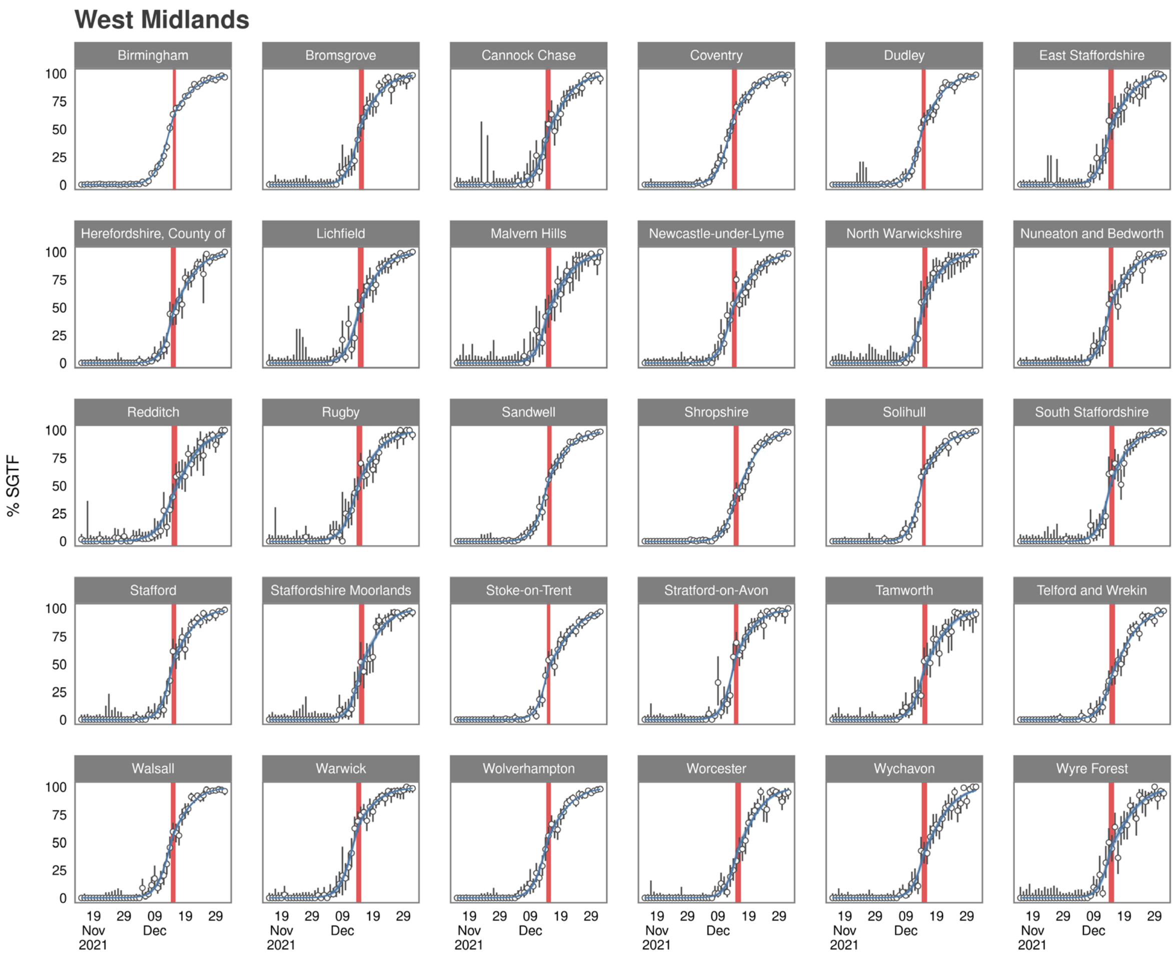

Figure S3: Model fit for LTLAs in the West Midlands.

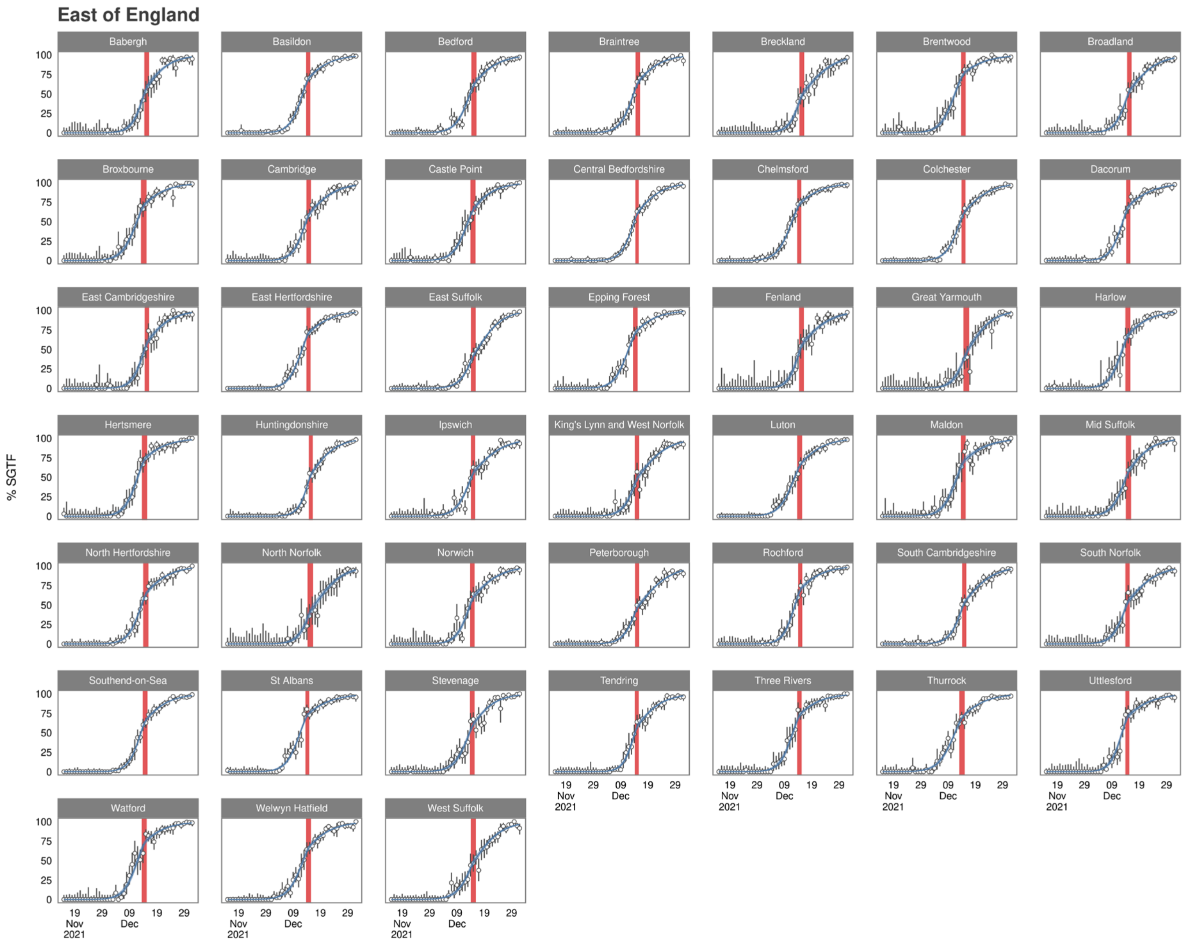

Figure S4: Model fit for LTLAs in the East of England.

North West

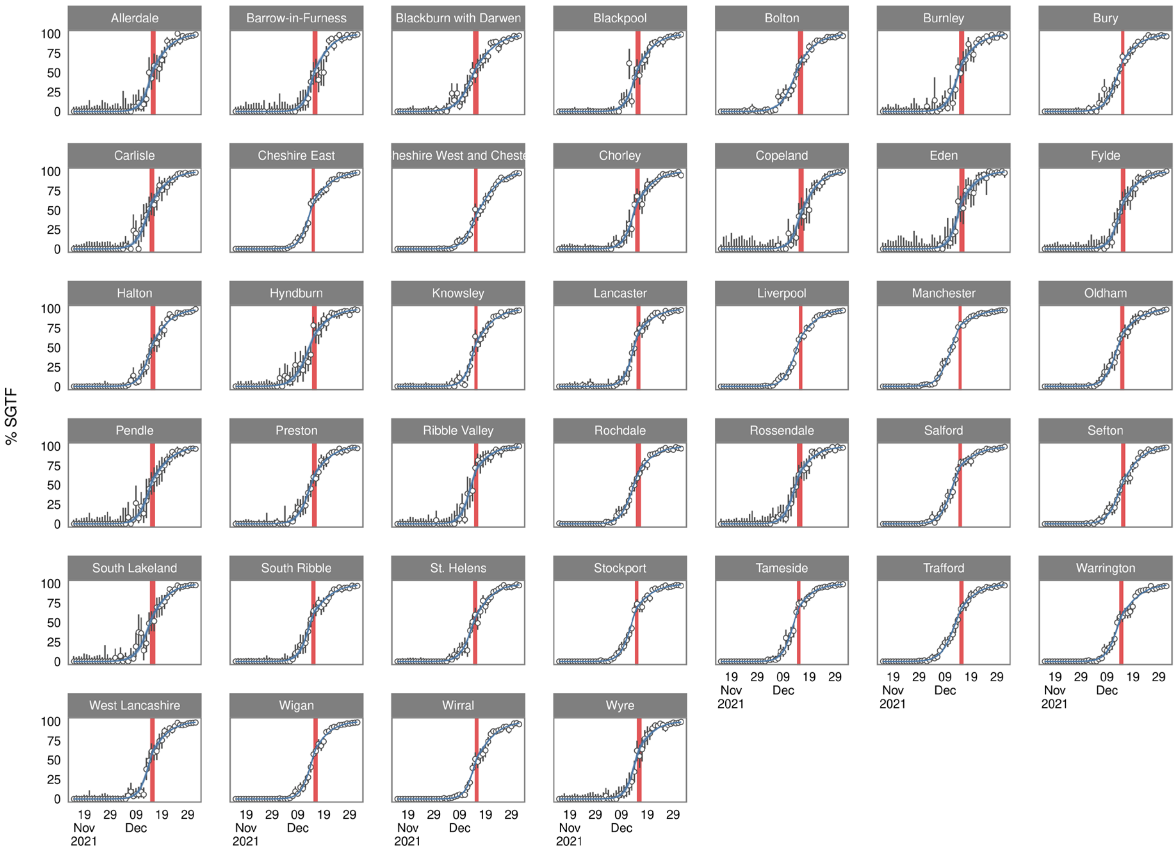

Figure S5: Model fit for LTLAs in the North West.

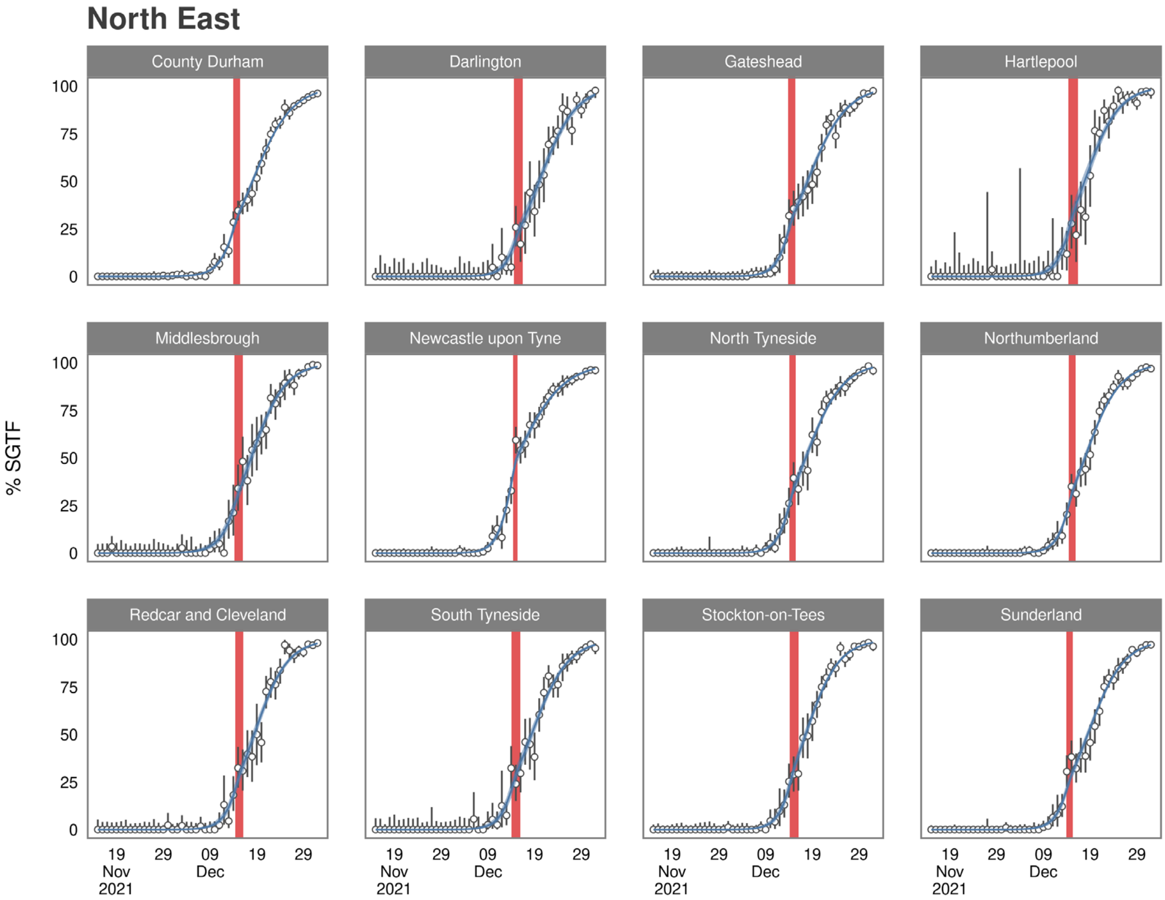

Figure S6: Model fit for LTLAs in the North East.

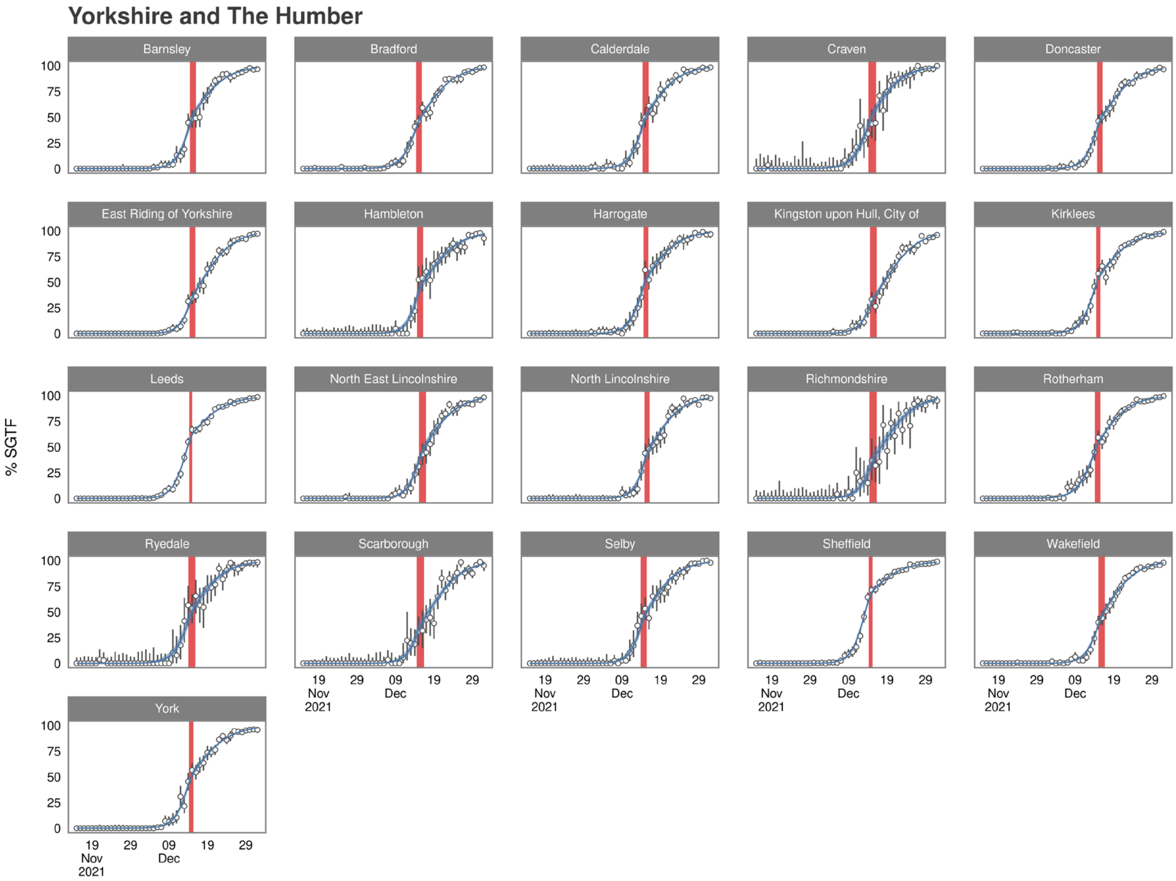

Figure S7: Model fit for LTLAs in Yorkshire and the Humber.

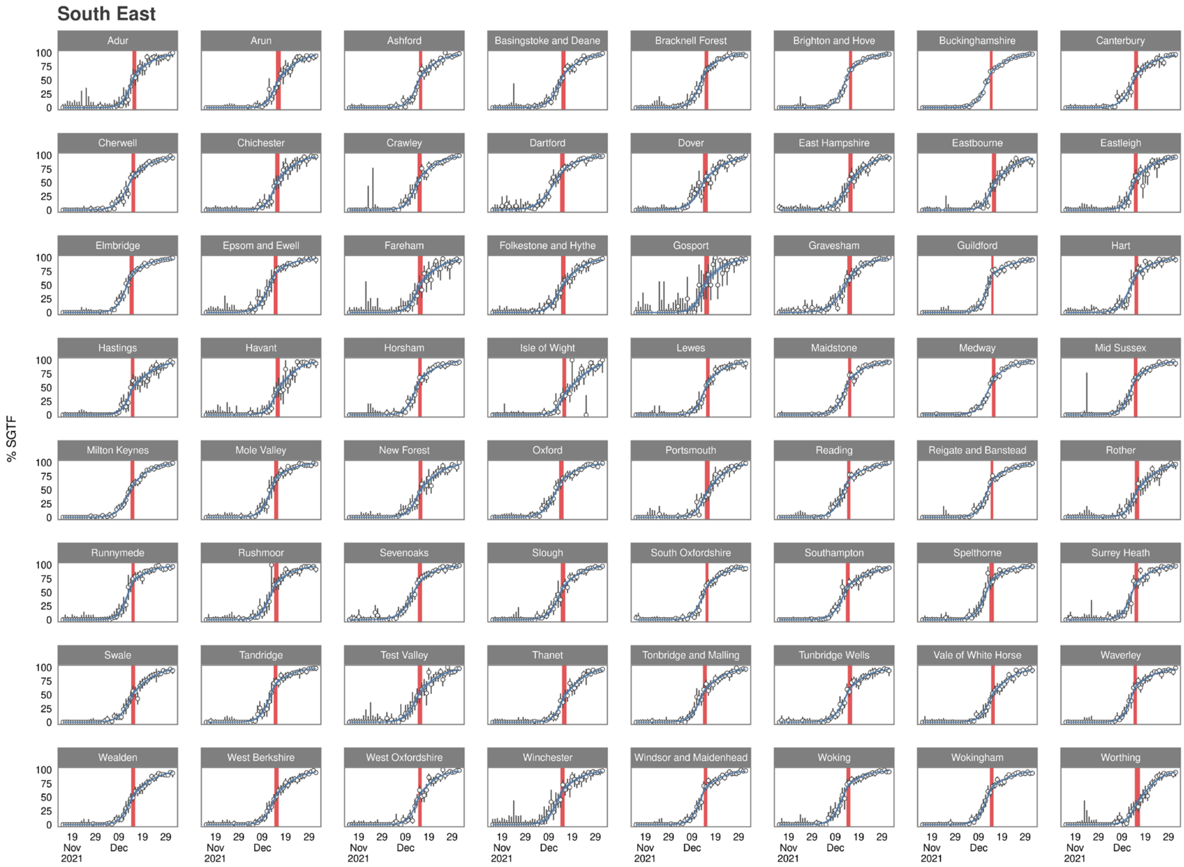

Figure S8: Model fit for LTLAs in South East.

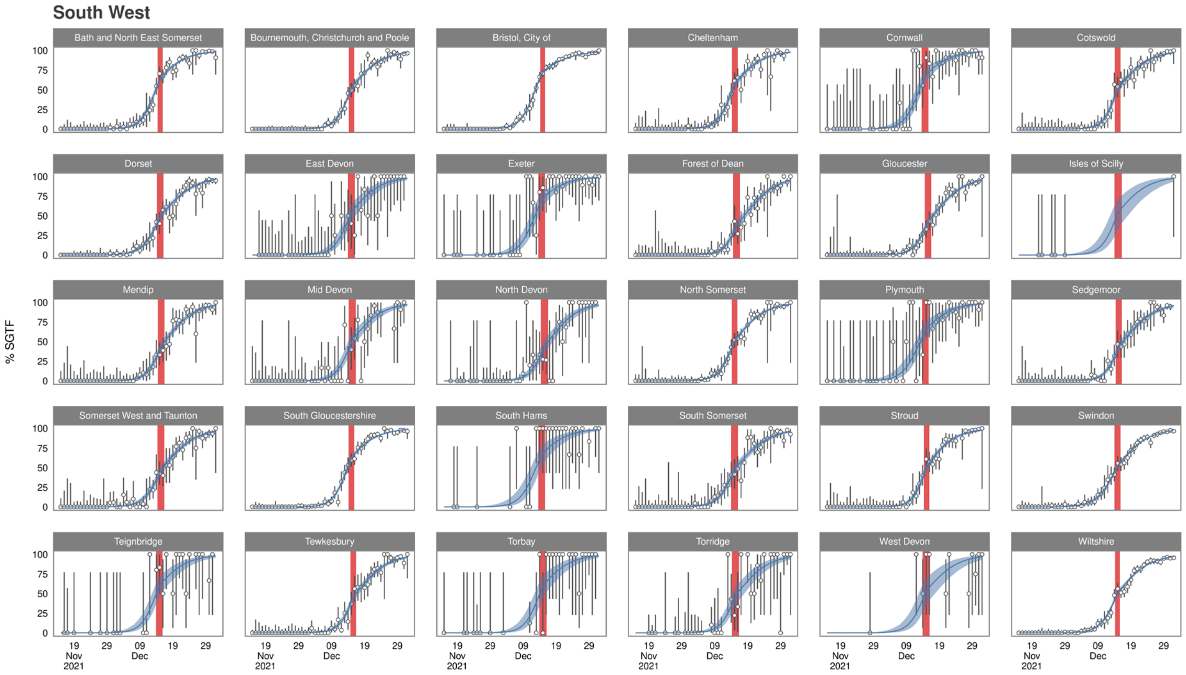

Figure S9: Model fit for LTLAs in South West.

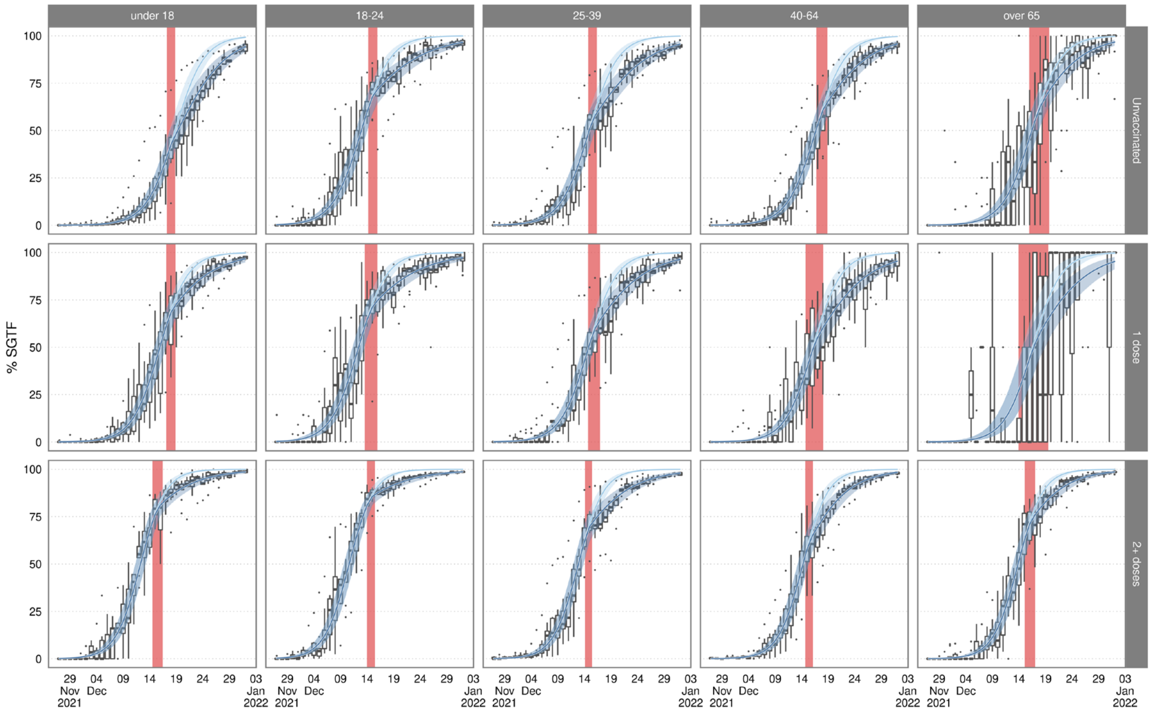

Figure S10: Model fit for the vaccine-stratified model, by age group. Boxplots show the regional proportions within each vaccine/age group.

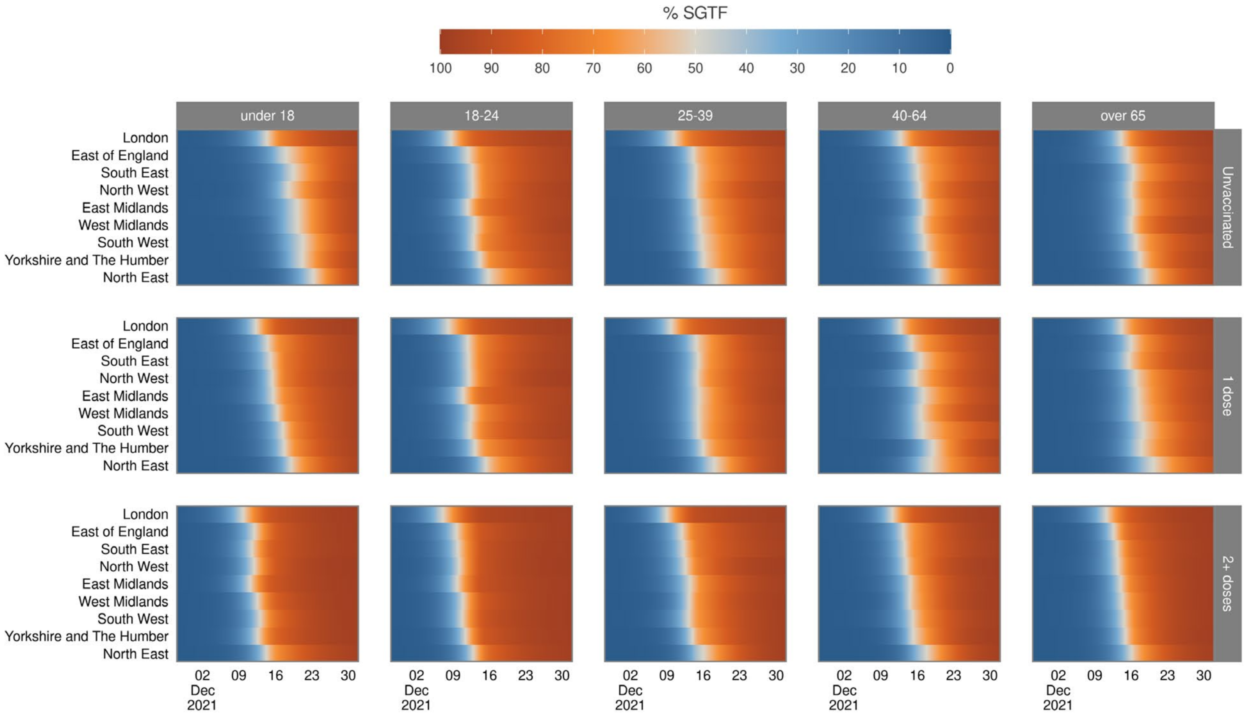

Figure S11: Surfaces showing the % of SGTF tests in each region.

Supplementary Table

PARAMETER	*PRIOR*	*LIMITS*
$\sigma_{\alpha^R}, \sigma_{\beta^R_{T<\tau}}, \sigma_{\beta^R_{T\geq\tau}}, \sigma_{\alpha^L}, \sigma_{\beta^L_{T<\tau}}, \sigma_{\beta^L_{T\geq\tau}}$	normal(0.1, 0.5)	$0, \infty$
$\sigma_{\tau^R}, \sigma_{\tau^L}$	normal(1.0, 1.0)	$0, \infty$
α	*normal(0, 0.5)*	$-\infty, \infty$
$\beta_{T<\tau}$	*normal(0.45, 0.5)*	$-\infty, \infty$
$\beta_{T\geq\tau}$	*normal(0.2, 0.5)*	$-\infty, \infty$
τ	*normal(30, 5)*	$0, \infty$

Table S1. Priors used for both models.
